# From source identification to preferential interventions: Determinants of a workplace mental health promotion program to control workplace stress among health care workers based on a qualitative study

**DOI:** 10.1371/journal.pone.0340575

**Published:** 2026-01-08

**Authors:** Masoume Zeinolabedini, Mohammad Esmaeil Motlagh, Alireza Heidarnia, Ghodratollah Shakerinejad

**Affiliations:** 1 Department of Health Education and Health Promotion, Faculty of Medical Sciences, Tarbiat Modares University (TMU), Tehran, Iran; 2 Department of Pediatrics, Ahvaz Jundishapur University of Medical Sciences, Ahvaz, Iran; 3 Health Education Research Department, Academic Center for Education, Culture and Research (ACECR)-Khuzestan, Ahvaz, Iran; Universiti Pertahanan Nasional Malaysia, MALAYSIA

## Abstract

Occupational stress among healthcare workers (HCWs) is increasing due to the development of healthcare services and increased occupational sensitivity. The aim of this study was to identify the determinants of a workplace mental health promotion program for stress management among HCWs. Data for this qualitative study were obtained through semi-structured interviews and focus groups. Data analysis was conducted using a directed content analysis approach, grounded in the PRECEDE-PROCEED model (PPM) and the Job Demands-Resources (JD-R) model. It was found that workplace stress has both individual and environmental (mainly organizational) origins. In the second phase of the program, ten subcategories were identified as effective factors in reducing employee stress in three categories: predisposing, enabling, and reinforcing factors. In the final step, in order to create changes and implement interventions, it was revealed that there is a need to formulate or revise managerial and structural policies in the organization and empower employees to improve interpersonal skills and manage workplace stress. Also, Non- occupational factors, Cognitive factors, Supervisory function, Personality characteristics, and Personal context of the employee were identified as new subcategories in the PPM to investigate work stress. Workplace stress has different sources and dimensions. Therefore, it is necessary to consider a combined and multi-level approach, including individual, social, organizational, and educational factors, for the mental health promotion program in the workplace. In this regard, the conceptual model proposed in this study can be a roadmap for researchers and employers to control the stress of HCWs and other employees.

## 1. Introduction

Workplace stress is defined as the response of individuals when work demands and pressures do not match their knowledge and ability, and their ability to cope is challenged [1]. Workplace stress is a global concern in all professions and all countries [2]. Studies show that workplace stress is a serious risk in health care settings [3,4] because the health care system is a sensitive public service provider. For example, the results of several studies showed that health care workers (HCWs) experienced high levels of stress during the coronavirus pandemic [5–8]. Long-term responses to chronic stressors at work lead to depression and burnout [9,10].

Despite the increasing number of studies and many methods of reducing workplace stress, it is still unclear how to design a mental health promotion program to develop successful and targeted interventions. Evidence shows that interventions based on mindfulness, education, and psychological therapies have been effective in reducing stress and burnout. However, to ensure psychological well-being in the workplace, attention to organizational and work-related factors is also essential [11]. While very limited evidence has been reported on organizational interventions [12], interventions should be preventive and promote positive leadership methods and other positive aspects of work [13].

Systematic reviews show that workplace stress is an important and complex yet integrated phenomenon. Therefore, researchers and managers are forced to use a multi-level approach to prevent and control workplace stress [14,15]. On the other hand, PPM can be a suitable guide and roadmap for designing and implementing workplace health promotion interventions to reduce workplace stress in various dimensions. Despite extensive research in this field, few studies have examined different dimensions of workplace stress [14,16]. Theories are facilitators of a researcher’s more profound understanding of what affects the health of individuals [17]. The PRECEDE-PROCEED Model (PPM) is a well-known and widely used planning model and has been used in numerous studies to implement workplace health promotion programs [18–20]. One of the notable features of PPM is its multilevel, ecological approach to health. Therefore, PPM can identify different dimensions of work-related stress. On the other hand, the PPM can also serve as a suitable guide and roadmap for designing and implementing workplace health promotion interventions to reduce workplace stress across various dimensions. To increase the effectiveness of the PPM in promoting mental health and stress management in the workplace, it is necessary to adapt this model specifically to workplace stress. The concept of workplace stress is closely tied to the work environment and organization and differs from the broader concept of stress. A qualitative study makes it possible to achieve this purpose. Also, since other models can be incorporated into the PPM, the Job Demand-Resource (JD-R) model [21] can be used to identify concepts related to workplace stress. The JD-R model is an excellent theoretical basis for assessing the mental health and well-being of workers and is used in various work settings.

This study aimed to identify determinants of a workplace mental health promotion program for stress management among HCWs, based on perceptions of employees and superiors and guided by the PPM.

## 2. Participants and methods

### 2.1. Study design

This qualitative study was conducted based on a directed content analysis approach. This approach is used when prior knowledge and theory about the target phenomenon are available and operational, and the researcher intends to test the prior theory under different conditions [22]. According to the purpose of the research, the PPM and the JD-R model were chosen to develop the research guide, analyze the data, and interpret the findings. This study focuses on phases 2, 3, and 4 of the PPM. To determine a framework for planning a public health program, these steps (the PRECEDE section) first identify factors influencing specific behaviors, then factors influencing change and maintaining change based on the identified factors, and finally, the facilitators and policies necessary for implementing a health promotion program [17]. An additional movie file shows this in more detail [see S1 Text]. The following research questions were followed:

1. Why do HCWs get stressed at work? (RQ1)
2. What is effective in reducing workplace stress? (RQ2)
3. What can be done to create change and intervention? (RQ3)

### 2.2. Participants

HCWs, supervisors, and managers were identified as relevant individuals to achieve the objectives of this study. Based on the structure of Iran’s health system, in primary health care centers, there are several HCWs and one HCW (foreman) who supervises and directs other HCWs. HCWs are responsible for providing health care services and controlling communicable and non-communicable diseases for all age groups covered by each health center. To achieve maximum diversity and experiences, sampling was conducted in a mixed manner [23]. Purposive and snowball sampling were used for HCWs. Inclusion criteria for this study were having at least six months of experience in primary health care centers as an HCW. First, a number of HCWs were selected from the list of employees of the Central Health Administration Department in two cities (based on work experience, type of responsibility, and geographical distribution of the workplace) and interviewed. Some participants introduced the researcher to a number of HCWs who were eligible for interviews (snowball sampling). Supervisors were selected by the purposive sampling method, and managers were selected by the convenience sampling method. Inclusion criteria were having at least 2 years of managerial work experience in the health department. The interviewer explained the study, the reasons, and the purpose of the study over the phone to the employees and invited them to participate in the research. Informed consent (written and oral) was obtained from all individuals before the interview, and assurance was given that the content of the interviews would be kept confidential.

### 2.3. Data collection

Semi-structured interview and focus group (FG) methods were chosen for data collection. Semi-structured interviews and focus groups were conducted between January 2020 and February 2021. An interview guide was designed based on the PPM and the workplace stress literature [see S2 Text]. To deeply understand the new information in each interview, that topic was included in the next interview. Exploratory questions were used when answers were ambiguous. All interviews were conducted at the participants’ workplace. Only the participant and the interviewer were present in the interview room. FGs took place in the meeting room of the organization. The average duration of interviews and FGs was 60 minutes. Interviews and FGs were recorded with a voice recorder, and each audio content was transcribed verbatim on the same day. Data collection continued until theoretical saturation [24] was reached. In other words, when no new information was found. One interview was repeated because of the need to clarify and explain an issue. For more certainty, two more interviews were conducted, but no new data was obtained. To prove the validity of the interviews and group discussions, the information was summarized and presented to the participants for reconfirmation.

### 2.4. Data analysis

Each transcript was read multiple times. In the preparation stage, based on the objectives of the study, the units of analysis were selected, and according to the general concepts, the meaning units were selected, and coding was performed. They were then compared based on similarities and differences and placed under categories and subcategories.

Since the directional content analysis approach was chosen for this study, a structured categorization matrix was developed based on the components of the PPM in MAXQDA software. The coding results were organized within the PPM framework. The JDR model was used to label subcategories that had semantic similarities with some concepts in the model, such as job demand and changes. In the analysis process, there were undecided texts containing meaningful codes. Despite their relevance to the research question, the undecided codes were not placed in any category. All remaining codes were grouped according to differences and similarities. Then new categories were named based on the general concept of the codes of each group.

Two researchers coded the transcripts. Then, based on the peer check strategy, two other researchers re-examined the codes. To increase inter-rater coding reliability, the codes and themes were repeatedly discussed by the team of authors, and only the themes that the research team had reached a consensus on were included in the results. The coding process involved analyzing field notes to gain depth and consensus. The coded interviews were also reviewed by participants to ensure the researchers’ interpretation of the data. Finally, a research psychologist in the field of qualitative studies was used to confirm the achievement of unbiased results and increase the accuracy of the categories. The research team tried not to merge their previous assumptions with the interpretations and extraction of the findings. In this regard, the techniques used were member check, interviewer training, and reminders.

### 2.5. Ethics statement

The study was conducted according to the guidelines of the Declaration of Helsinki and approved by the Ethics Committee of Tarbiat Modares University (the ethics code number: IR.MODARES.REC.1397.032).

## 3. Results

HCWs from eighteen primary health care centers participated in this study. Data came from 11 semi-structured interviews, 2 focus groups with HCWs, and 2 focus groups with supervisors and managers. The demographic information of the sample is presented in Table 1. The average age of HCWs and superiors was 33.7 and 41.6. The average job experience of HCWs and superiors was 7.2 and 11.9. All participants in the study had university education in health-related fields of study. See detailed participant profiles in S3 Table.

**Table 1:** Social-professional characteristics of health care workers and superiors.

Social-professional characteristics		N = 32
**Gender**	Female	29
	Male	3
**Profession**	Health care worker	21
	Manager	2
	Supervisor	9
**Foreman**	Yes	16
	No	5
**Parent**	Yes	11
	No	10
**Marital status**	Married	15
	Single	6
**Employment Contract**	Permanent contract	8
	Fixed-term contract	13
**Education**	Bachelor’s degree	21
**Educational field**	Public health	13
	Midwifery	8

**Fig 1:**
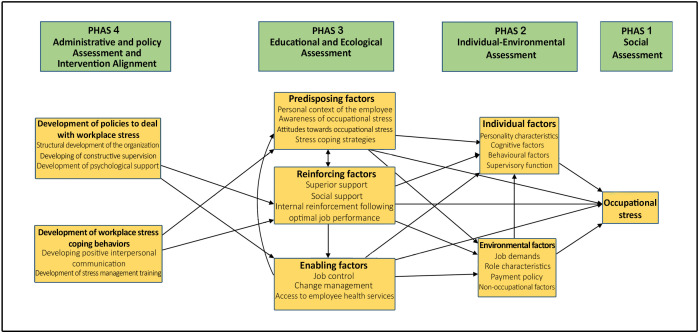
The conceptual framework for promoting mental health in the workplace, based on the PPM, to reduce workplace stress.

The overall results of the study are presented in Fig 1 as a conceptual framework. The findings are reported in the order of research questions and based on the steps of the PPM. The data were placed in 23 subcategories and 7 categories and three phases of the PPM, including individual-environmental assessment, educational and environmental assessment, and administrative and policy assessment and intervention alignment.

### 3.1. Individual-environmental assessment

The results of this stage are focused on research question RQ1. In fact, the first phase of the Workplace Mental Health Promotion Program identified stressors among healthcare workers. The results were divided into two categories: personal and environmental stressors (see Table 2).

**Table 2:** Categories, subcategories, and some codes of individual-environmental assessment.

Category	Subcategory	Code
**Environmental factors**	Job demands	Workload Responding to different units of the organization Client service challenges Lack of job security Job content
	Role characteristics	Role conflict Role ambiguity Role load
	Payment policies	Injustice in certain payments Lack of performance-based payment Payment based on employment status
	Non-occupational factors	Family issues Financial worries Being the parent
**Individual factors**	Personality characteristics	Anxious personality Idealism Work conscience
	Cognitive factors	Beliefs about the organization’s climate Perceiving injustice Expectations from Self
	Behavioral factors	Interpersonal communication in the workplace Employee job performance
	Supervisory function	Unfair performance of supervisors Monitoring methods

#### 3.1.1. Environmental factors.

Environmental stressors were beyond the control of the employees, and their origin was the organization’s policies, job-related conditions, and environmental factors not related to the job.

**Job demands.** It was observed that job demands and job-related factors are often mentioned as stressful factors. All participants considered increasing workload as an important source of stress in HCWs. One of the supervisors said:

*The biggest problem for HCWs, especially foremen, is the high workload and scope of work. They say, “We are confused about what to do.” Now that the coronavirus pandemic has occurred, their work has multiplied (P23-FG).*

Also, HCWs had to be accountable to different supervisory units; they tried to satisfy the supervisors. Participants mentioned the pressure of working with clients because the clients were different in terms of culture, education, beliefs, values, and income. Therefore, HCWs were sometimes faced with unreasonable and impossible expectations from clients. An HCW told the interviewer:

*Our clients have different social statuses, moral characteristics, and expectations, but they all receive the same service, and they may not be satisfied with our services and may react with inappropriate behaviour (P3).*

Lack of job security and uncertain job prospects were the most common concerns of HCWs with fixed-term contracts. Some of them had to endure the problems and not protest to avoid the cancellation of the contract.

**Role characteristics.** An anxious situation for HCWs is when they find the organization’s expectations to conflict with their own or the client’s beliefs. One HCW said about the experience of being severely warned by a superior after acting contrary to instructions:

*Why did my work lead to a supervisor’s notice? I considered the spiritual aspect of that work, and I did charitable work. Why did this end?!! (P6)*

Lack of transparency in some career goals and expectations has led to confusion and the impossibility of planning for HCWs. Moreover, all participants identified multitasking as a key problem for HCWs. They believed that managers focus on HCWs. Health care workers are at the forefront of most primary health care and new services. Another significant source of stress among foremen was having two different roles at the same time (foremen and HCW). One of the foremen said about the role overload:

*Our workload is too much; our stress is too much. I worry about work when I am at home; they sent me some letters today, and I have to implement them... (P4).*

**Payment policies.** The participants attributed one of the reasons for the mental harassment of HCWs to some payment approaches. For example, the surcharge was only for services that could be registered on the electronic health website. Participants, especially HCWs with fixed-term contracts, considered payment based on the type of employment contract as one of the factors of stress. Employees with fixed-term contracts had the same duties as other employees, yet they were paid less and felt it was unfair. One of the superiors said:

*They are upset and say, “We did all that work, but others got the rewards! How is that possible?” (P27-FG).*

**Non-occupational factors.** Beyond workplace environmental factors, non-work factors like parenting, family care, and financial problems also put pressure on HCWs. An HCW with two children said:

*I use break time for the nursing mothers’ law, so I work one hour less than my colleagues and am one hour behind them (P1).*

#### 3.1.2. Individual factors.

It was found that beyond the external environment, the personal characteristics and performance of employees, superiors, and managers can lead to stress in HCWs. These factors put pressure on HCWs directly and indirectly.

**Personal characteristics.** Employee personality traits were found to be effective in adopting behaviors that create stress or reinforce stressful situations in the workplace, such as extreme idealism. An HCW said:

*The personalities of individuals are influential; people with anxious personalities, even though they know they are performing their duties properly, still become anxious when they threaten others (P3).*

Several healthcare workers were stressed due to some of their beliefs, such as the belief that some superiors insist on finding weaknesses in HCWs’ performance or HCWs’ perceptions of their colleagues. An HCW, after receiving a warning from a superior, expressed the cause of her stress as follows:

*I didn’t feel punished; I felt a sense of pride and superiority over my colleagues, and I was annoyed (P6).*

**Cognitive factors.** A person’s processing and understanding of what happens at the workplace was identified as an effective factor in stress. It was found that the health care worker’s dissatisfaction with her job performance leads to self-blame and stress, and this is due to not meeting the expectations that she has of herself. One participant stated that she wanted everyone to be satisfied with her. Her inability to please others made her anxious.

How to understand some job characteristics was a mental concern for HCWs. Some HCWs were stressed due to some of their beliefs, such as the belief that some superiors insist on finding weaknesses in HCWs’ performance or HCWs’ perceptions of their colleagues. Participants stated that some managers have little respect and value for HCWs working in primary health care facilities. An HCW said:

*My job has a great impact on improving the health of society. I love my job and my duties very much. I expect my job to have a valuable place in the organization; however, this is not the case, and this is painful (P8).*

**Behavioural factors.** Participants cited the behavior of HCWs, colleagues, supervisors, and clients in the workplace as a significant and common source of stress. HCWs and supervisors repeatedly emphasized that workplace harassment leads to increased psychological stress among employees. This study found that some severe emotional distress is due to mistreatment, tension, and poor respect in workplace relationships. A superior said about the tension between a supervisor and an HCW:

*That supervisor had a strong conflict with the HCW in front of other colleagues and clients. That supervisor had a strong conflict with the HCW in front of other colleagues and clients. The HCW said, “After this happened, I am very embarrassed among the people of this village” (P29-FG).*

On the other hand, HCWs mentioned the impact of the employee’s reaction and skill in different communication situations. An HCW said about the stress of talking to a client:

*When the alcohol pad runs out, I stress about how to convince the client that we cannot vaccinate and that he/she should come again (P20).*

Some participants stated that incomplete performance of tasks, lack of daily planning, and carelessness in performing sensitive tasks by HCWs cause anxiety and distress.

**Supervisory function.** The performance of supervisors was prominently mentioned as an influential factor. Participants believed that unfair performance by managers and supervisors causes feelings of dissatisfaction. For example, an HCW had been selected as an exemplary employee several times, but managers had promoted another employee who was performing poorly. She said:

*They acted very unfairly. What are the rules for career promotion? What are the criteria? This situation is very disturbing to me (P10).*

Superiors and HCWs stated that monitoring methods are one of the causes of anxiety. In addition, supervisors’ poor skills in supervision, training, and problem analysis created stressful challenges for HCWs. The challenge that most participants indicated was the way superiors reacted to an employee who was performing well compared to an employee who was performing poorly. Sometimes an employee who worked well was given more work. A manager described this issue as follows:

*Assigning work to a competent employee is the right approach for the organization, which should be based on meritocracy. But the problem is that the assignment of work and benefits is not proportionate. On the other hand, out of compassion, we don’t want the weak employee to lose his or her job. Nonetheless, we don’t have a solution to deal with this employee, so we leave the job to others, and this is a wrong solution (P32-FG).*

### 3.2. Educational and ecological assessment

The findings of this phase answer the second research question (RQ2). In other words, the second phase of the workplace mental health promotion program explored the predisposing, enabling, and reinforcing factors for creating change in various aspects of workplace stress (see Table 3).

**Table 3:** Categories, subcategories, and some main codes of educational and ecological assessment.

Category	Subcategory	Main Codes
**Predisposing factors**	Personal context of the employee	Self-confidence Perceiving self-worth Belief in having a worthwhile job Self-monitoring
	Awareness of occupational stress	Awareness about stress management Awareness of the causes of stress
	Attitudes towards occupational stress	Perceived beliefs about the cause of stress Perceived beliefs about of stress management
	Stress coping strategies	Relaxing strategies Problem- focused coping Emotional self-control Maladaptive strategies
**Enabling factors**	Job autonomy	High responsiveness Responsibility without authority Being forced to do certain things without the right to choose Freedom to plan daily tasks
	Change management	Forced transfer health care workers Poor training support for new instructions Frequent changes
	Access to employee health services	Lack of special health care services for health workers No allotment of medical leave for health care
**Reinforcing factors**	Superior support	Encouraging Health Care Workers Superior confidence in the ability of employees Provide opportunities for employees to express their demand Understanding employee concerns
	Social support	Family supportive behavior Colleagues emotional support Colleagues’ participation Positive client’s feedback
	Internal reinforcement following optimal job performance	The pleasant feeling of helping people improve their health A sense of self-satisfaction following a favorable job performance Sense of competence

#### 3.2.1. Predisposing factors.

Predisposing factors cause behavior related to stress control, and in other words, they precede the behavior. Including personal potential and context that can contribute to reducing or increasing stress.

**Personal context of the employee.** People’s confidence in their own abilities to perform job duties and overcome psychological pressure was identified as a personal context in stress control. Furthermore, it was found that when HCWs realize they are useful and valuable in providing others’ health and consider their job valuable, they experience a positive and energizing feeling. An HCW believed:

*Everything starts with us… I am the one who has to determine whether this screening is positive or not; I am the one who determines whether this child has a developmental disorder or not. This is enjoyable… that is, I am important. It even becomes clear that my negligence may have harmful consequences (P6).*

For some HCWs, it was very significant for their efforts to be seen by supervisors and others. HCWs viewed failure to do so as annoying and disrespectful. On the other hand, HCW’s attitude towards monitoring can be an effective or a hindrance factor for managing workplace-related stressors. One of the participants in the focus group said:

*A midwife should follow up on a high-risk pregnant mother. It is a very sensitive task. If we are not careful, the lives of the mother and her child will be in danger. In these situations, monitoring and pressure from the organization are necessary*... *(P21).*

**Awareness of occupational stress.** According to participants, awareness of stress and its causes help them perform better in stressful situations. An HCW commented on how awareness can help reduce stress:

*When my colleague gets angry, I know why she is angry; knowing this reduces my stress (P6).*

**Attitudes towards occupational stress.** Identifying employees’ attitudes towards occupational stress is important for effective interventions and creating stress-controlling behaviors in the workplace. Some HCWs believed that the organization is the cause of work stress, while others pointed to the importance of the individual’s role. Some participants believed that stress can be controlled. They considered their role as necessary to prevent the physical and mental effects of stress. On the contrary, some participants believed that their stress is involuntary and they cannot control it. An HCW who experienced a lot of stress at work believed:

*Maybe it’s my fault that I can’t control my stress; maybe I get obsessive-compulsive (P8).*

**Stress coping strategies.** HCWs used individual strategies to reduce tension and obtain relaxation in facing stressful situations and factors. Some participants used strategies such as exercising, traveling, listening to music, and reading personal development books to reduce stress. Other strategies were used to solve problems caused by the job and workplace and were perceived as very useful, such as being flexible with clients, changing work style, and increasing recognition of colleagues and superiors. One of the HCWs spoke about the importance of coping with stress:

*When I prioritize improving a job weakness, my stress goes down (P4).*

HCWs sometimes had emotional self-control to cope with work stress. For instance, they may use spirituality, engage in positive self-talk, remain silent in the face of unpleasant behaviors, or lighten the mood with humorous topics. On the other hand, some HCWs had maladaptive strategies to reduce pressure, such as negative reactions to job performance, selective performance of tasks, anger, mental conflict, taking medicine, and finally, a desire to change jobs. One of the supervisors said about the reaction of HCWs when faced with high work pressure:

*When they are under pressure, they do a part of the work and leave the rest (F29-FG).*

#### 3.2.2. Enabling factors.

Enabling factors can directly or indirectly allow a supportive motivation, behavior, or policy to be realized to control work stress, so they should be changed if needed.

**Job autonomy.** If HCWs feel that they do not have control over important aspects of their jobs and are faced with a volume of demands and limitations, the conditions for the emergence of stress are provided. The participants emphasized that supervision of supervisors and accountability to senior managers are high, and this causes a feeling of high organizational control and low independence. An HCW said:

*It’s awful; it makes me feel so bad. It’s like we don’t know anything, and everyone has to tell us what to do (P6).*

The participants, especially the foremen, stated that not having the necessary authority for the assigned responsibility leads to stressful challenges for them. On the other hand, HCWs believed that freedom of action in the speed and order of performing tasks leads to relaxation and better management of things. One HCW said something impressive:

*When my work goes according to my plan and there is nothing left for me, I am not stressed (P5).*

**Change management.** Occupational and organizational changes (such as transferring HCWs, adding new programs, and changing guidelines) have created pressures and challenges for HCWs. The participants mentioned examples where the unexpected sending of a transfer letter and forcing the HCW to change the workplace had caused a disturbing shock to the HCW and the foreman. On the other hand, the importance of education was noted for changes. This is while the participants mentioned anxiety and high workload due to poor educational support. An HCW commented on the need for timely training on changes in the vaccination registration process:

*If they had taught us from the beginning how to properly register vaccines on the health site, we wouldn’t be facing problems now and wouldn’t have had to correct many errors in a very limited time (P5).*

**Access to employee health services.** While access to health services improves employee health and well-being, the participants claimed that not only is there no special medical service for employees in their organization, but they experience concerns such as spending time on accessing health services and staying in the appointment process. Regarding access to a mental health professional, an HCW said:

*We have a psychologist in our center (for clients). She comes and talks to us when we have a problem. That’s great (P2).*

#### 3.2.3. Reinforcing factors.

Factors that lead to the pursuit of stress reduction strategies and good job performance and provide continuous motivation, reward, and encouragement to maintain them.

**Superior support.** Participants believed that managers and superiors who encourage and trust HCWs reduce the mental stress and distress of HCWs and increase job motivation. An HCW who was monitored several times by the ministry said:

*When they come from the ministry for inspection, the manager can be effective in reducing stress. For example, our manager comforted us and said, “Don’t worry, you are doing your job very well,” and this made us feel safe (P10).*

Furthermore, according to the participants, HCWs’ motivation to correct job weaknesses increased when supervisor support and encouragement were felt in their monitoring feedback. It was reported that providing opportunities for HCWs to express their demands and supporting them in work and non-work problems and concerns creates a pleasant feeling of security and peace for HCWs. In contrast, one supervisor said about the need to support employees who are in trouble:

*We had an HCW who was involved in a serious insurance problem. She even thought of killing herself and her child. She had referred to the central office many times, but nothing was done for her... She said, “There was no one to hear my words...” (F27-FG).*

**Social support.** On the other hand, most of the participants mentioned the importance of emotional support from colleagues and family in facing job pressures. It was also mentioned that when the workload is high, the support and participation of colleagues are effective, and sometimes it has a two-way effect. A foreman said about the consequences of helping her colleague in a critical situation:

*Every day, she leaves a note on my desk: “Thank you for being you; you are the best.” I kept all those notes; those notes have positive energy and give me motivation and peace (P16).*

Some HCWs described receiving positive feedback from clients as one of the motivating factors at work. One HCW said:

*When the client is satisfied, just one word says, “Have a good future,” and I feel very satisfied (P15).*

**Internal reinforcement following optimal job performance.** All HCWs experienced a pleasant feeling of satisfaction and peace after solving clients’ health problems or performing their job duties satisfactorily. An HCW said of her feelings:

*When a mother can’t breastfeed her baby, the baby rejects the mother’s breast. I help the baby accept the mother’s breast. It’s an excellent feeling for the mother and me because I managed to save the mother from anxiety and discomfort (P5).*

### 3.3. Administrative and policy assessment and intervention alignment

In the third phase of the program to create changes and implement health promotion interventions in the workplace with the aim of reducing stress (findings of the third research question), participants’ preferences were divided into two categories: Development of policies to deal with workplace stress and Development of workplace stress coping behaviors (see Table 4).

**Table 4:** Categories, subcategories, and some main codes of administrative and policy assessment and intervention alignment.

Category	Subcategory	Main Codes
**Development of policies to deal with workplace stress**	Structural development of the organization	Job redesign Change in executive management performance Job skills training Performance based payment Determining the framework of job promotion Employee participation in decision making
	Developing of constructive supervision	Supportive monitoring and positive guidance Focused supervision on problem solving Improving supervisory skills Improving how to respond to poor performance
	Development of psychological support	Strategies to increase the motivation of HCWs Supporting employees who are in trouble Creating mental health services for employees
**Development of workplace stress coping behaviors**	Developing positive interpersonal communication	The importance of friendly relations and mutual respect in the workplace Improve the behavior of supervisors during monitoring Getting feedback from employees about supervisors
	Development of stress management training	The need for stress management training Stress management training through peer to peer

#### 3.3.1. Development of policies to deal with work stress.

To implement, develop, and strengthen effective organizational interventions, it is essential to establish policies and laws, including the creation of new reforms and laws in the organizational structure.

**Structural development of the organization.** The participants suggested redesigning roles through specialized division of work, assigning tasks according to individual characteristics and capabilities. A manager said:

*Each person has special abilities and conditions. I must consider this important point in selecting my employees. For example, one person is useful for training, another is useful for executive work, and another is suitable for supervision (F31-FG).*

Managers and supervisors considered it important to make changes in the organization’s executive management process, including modifying some work processes to reduce workload and improve job skills training. Furthermore, they believed that the allocation of payments should be based on workload and job sensitivity. To organize the job promotion system and eliminate the feeling of being oppressed among the HCWs, the necessity of setting up a specific framework for job promotion was emphasized. Moreover, the participants of this study considered it important to involve HCWs in carefully examining the stressful factors of the work environment, discovering efficient solutions, deciding how to make changes, and handing over responsibilities.

**Developing of constructive supervision.** According to the participants, especially the supervisors, a review of supervision techniques is necessary to reduce stressful supervision. In this regard, issues such as conducting supervision based on problem-solving, improving supervisors’ supervisory skills, and improving methods of dealing with poor performance were mentioned. Regarding how to deal with a weak employee, a supervisor said:

*We need to understand what the problem is; maybe there is a problem with the work process, and maybe we have to solve that problem ourselves (F25-FG).*

**Development of psychological support.** Participants found it useful to provide psychological support to HCWs through positive support and guidance, support in dealing with work and non-work problems, and periodic mental health check-ups. An HCW said in a focus group:

*It is enough for the supervisors to see my positive points; this gives me a lot of peace (P17).*

HCWs wanted to develop motivational factors in the workplace. Supervisors believed that, in addition to monetary rewards, non-monetary rewards were also effective, such as verbal encouragement and providing the employee with a desired job position.

#### 3.3.2. Development of workplace stress coping behaviors.

Although many participants mentioned solutions related to organizational changes, in addition, they believed that behavioral and educational interventions are also useful for reducing the psychological pressure on employees.

**Developing positive interpersonal communication.** Participants noted the importance of promoting friendly communication and mutual respect between co-workers and supervisors to reduce tensions and advance career goals. A foreman said:

*Instead of supervisors saying, “It’s terrible,” they can say, “If you do these things, it will be better, and you can reach the expected results by the end of the year” (P11).*

**Development of stress management training.** HCWs emphasized the importance of learning how to manage stress and requested the implementation of training programs in this area. They also mentioned their role in developing and sharing new experiences and skills. An HCW said:

*When we all received training and learned those skills, I realised how to reduce my stress, and I can help my colleagues reduce theirs by training them, and vice versa (P6).*

## 4. Discussion

This qualitative study provides a conceptual and three-stage framework for the determinants of a mental health promotion program for stress management, from identifying the source to designing an intervention among HCWs. In this regard, it is necessary to first identify the sources of workplace stress. Then, factors affecting change and facilitators of implementing a mental health promotion program in the workplace are determined. The findings are based on the perception of employees, supervisors, and managers of workplace stress among HCWs working in primary health care centers. In this regard, the PRECEDE-PROCEED planning model was used as the basic model, along with some concepts of the JD-R model.

According to the results of this study, workplace stress generally has environmental and individual roots. With this in mind, job demand is an important environmental factor. It was observed that high job demand has an effect on increased psychological strain, stress, and poor job performance [25,26]. Also, a negative relationship between demand and public service motivation has been reported [25]. According to our findings, HCWs have a high workload due to their extensive duties. Furthermore, they have to answer to multiple high-level organizational units. As a result of this volume of demands, significant confusion and anxiety are caused for HCWs. In this study, the pressures were exacerbated by the outbreak of coronavirus disease.

The importance of the characteristics of the role has been noticed and confirmed by many studies [26]. We found that there is role overload in foremen. In addition to the duties of an HCW, foremen must also perform administrative and organizational tasks, so they experience multitasking and role pressure. This can be a special job condition for foreman HCWs in Iran. Similar to previous studies [27], it was found that role conflict is a stressful factor. Role conflict is a situation in which an HCW is required to follow a set of conflicting job demands and values.

Furthermore, our findings indicated that poor payment policies lead to the perception of injustice among HCWs. A review study found that pay is not a motivator, but the unfairness of payment is annoying [28]. It was found that, in addition to the factors related to the work environment, sometimes the aspects related to the social role (wife, parent, and child) can also put pressure on the HCW at the workplace. In this regard, scientific evidence shows that sometimes people are psychologically victims of managing the relationship between work and home [29].

Previous studies have emphasized the need to conduct multi-layered research on workplace stress [30,31]. In line with this issue, one of the prominent findings of this study was the identification of the impact of individual factors alongside environmental factors. It was found that the personal characteristics of HCWs can be effective in the occurrence of workplace stress. Other evidence also argues that personality influences stress assessment and coping [32,33]. One of the most important individual differences is people’s different interpretations of different situations and conditions [34]. In this regard, a significant result of this study is the identification of the cognitive function of HCWs in the event of stress. Employees experience severe mental pressure when they perceive what is happening in the organization as cruelty and injustice and have negative beliefs about the organization, superiors, and colleagues. In addition to the mentioned factors, researchers stated that the quality of relationships between employees is associated with increased work commitment and reduced stress [35,36]. The results of our study similarly show the pressure caused by undesirable behaviors such as harassment, verbal abuse, and humiliating behavior, especially among clients and supervisors.

Another prominent result in this study was the identification of supervisors’ performance as an important and stimulating individual factor in the emergence of stress. The weak performance and skills of the supervisors in the field of monitoring and supervision for the HCWs result in unfair judgment, pressure, and threats. To our knowledge, very limited studies have investigated the role of supervisor performance in health care workers’ stress.

To design a health promotion program to control stress in the workplace, it is necessary to pay attention to the effective and facilitating factors, taking into account the various identified causes. We found that in addition to the origin, the factors affecting the reduction of workplace stress are also multidimensional. One of the specific results of this study is the identification of personal context as a predisposing factor in controlling workplace stress. Previous studies have pointed out the importance of the role of self-efficacy, self-esteem, and optimism in work-related well-being [37]. Bandura defined self-efficacy as a person’s confidence in his or her ability to organize and implement a certain action to solve a problem or perform a task [38]. Other evidence reported a negative correlation between self-efficacy and stress control [39]. Additionally, HCWs with low self-esteem are more likely to experience high stress [40]. In our study, it was also observed that HCWs who understand the value of themselves and their work and have confidence in their abilities to manage work have high job satisfaction and motivation. On the other hand, employees’ belief in the impact of their job performance on the emergence of stress and their desire for self-monitoring can lead to addressing weaknesses in job performance and reducing stress.

Employees’ knowledge and attitude toward stress at work is another important aspect for implementing a targeted stress reduction intervention at the individual level, especially for training related to stress management. Several theories focus on how beliefs relate to successful behaviors [41]. This is particularly important as a locus of control and a predictor of job-related attitudes and behaviors [42,43]. According to locus of control theory [44], believing that others are responsible for creating an outcome, as a source of external control, predicts avoidance behavior in the face of stress. On the other hand, the internal locus of control is related to help-seeking and positive thinking [45]. Similarly, it was observed that HCWs who perceive the source of stress as internal factors have different attitudes, such as the importance of their role and the ability to manage stress.

Consistent with other qualitative studies [35,46], the participants of this study tended to use problem-focused emotional self-control and maladaptive strategies to cope with stress. In the experience of HCWs, problem-focused strategies are more useful in reducing stress. In a previous study, HCWs during the COVID-19 pandemic often used a problem-solving coping style [47]. A study by Rollin and colleagues found that HCWs who used a problem-focused coping style had low perceived threat and high perceived control in response to the COVID-19 pandemic [48]. Meanwhile, similar to our findings, the study by Bakker & de Vries reports that employees turn to maladaptive self-regulation strategies when faced with increased job pressure [49]. If they do not achieve this goal, they will experience disappointment and even depression.

Neglecting enabling factors can act as a barrier to achieving the intervention goal. Most studies have identified job autonomy as a moderating factor of stress in the workplace [28,50]. In this study, it was observed that high levels of organizational control reduce independence and increase psychological stress. Job and organizational changes are a natural feature of work, but if change management is poor, it can lead to resistance to change, feelings of job insecurity, and anxiety [51]. Similarly, our findings confirm this issue.

The reinforcing factors for creating and maintaining motivation and stress-reducing behaviors in this study have individual, social, and organizational aspects. According to our findings, when HCWs are supported by managers and supervisors, they experience greater work commitment and peace of mind. Receiving feedback from a manager can boost performance and motivation, and the lack of it can lead to stress [52]. It was found that if performance feedback is accompanied by guidance and encouragement, it can motivate and improve performance; otherwise, it can be a key stressor. In addition, the support of colleagues in the form of work partnerships and emotional support is also important in managing stress. Foremen, who had different administrative roles, reported poor support from colleagues, increased workload, and psychological stress. It seems that increased responsibility can affect the importance of Colleague support. Similarly, the findings of several studies emphasize the importance and positive impact of organizational and social support on improving workplace mental health and job motivation [6,30,53].

Moreover, the participants of this study repeatedly mentioned the understanding, importance, and impact of internal rewards, such as personal satisfaction from completing an activity and gaining a sense of competence. Other studies have acknowledged the importance of intrinsic rewards in increasing motivation and improving job performance [54–56]. Self-determination theory supports a number of new categories in this study, for example, employee personal context and internal reinforcement. According to the theory of self-determination, all employees have three psychological needs (competence, autonomy, and communication) that increase intrinsic, extrinsic, and evolved motivation and ultimately increase well-being and performance quality [57].

A systematic review was conducted to investigate the effectiveness of individual and organizational interventions for managing workplace stress in HCWs. Catapano et al. compared three intervention methods, including interventions based on cognitive-behavioral therapy, relaxation techniques, and organizational-level interventions. The results of that study showed that it is not possible to definitively select the best intervention method [15]. Our study highlights that the sources of workplace stress and the factors influencing its reduction are multidimensional, so a mixed intervention guide is essential to manage stress among HCWs. At the organizational level, it is essential to develop, modify, and refine organizational policies, management, and supervisory approaches. Organizations can minimize organizational control by redesigning jobs and empowering employees for job autonomy [58,59]. In addition, consulting with employees about potential job changes and providing them with the opportunity to influence how these changes are implemented can help prevent stress. Also, creating transparent, performance-based frameworks for career advancement and fair pay has been effective. Similar to a review study, we found that constructive supervision not only protects the individual from the negative effects of stress but also increases the productivity and motivation of employees. In this regard, empowering managers and supervisors with a focus on improving supervisory and management techniques and skills seems important. We also found that organizational supports need to be incorporated into organizational-level interventions. For this purpose, assigning a mental health counsellor and legal counsel and developing motivational management to support HCWs can be useful. In organizational interventions, utilizing transformational leadership theory can be a worthy choice. Transformational leadership will be able to create a positive work environment, a sense of social justice, job satisfaction, organizational citizenship behavior, coping with the negative consequences of workplace stress, job motivation, and a sense of optimism [60]. This is fully consistent with our findings in the third stage of the conceptual model of promoting mental health in the workplace. This theory is fully consistent with our findings in the third stage of the conceptual model of promoting mental health in the workplace.

On the other hand, the findings of this study highlight the importance of paying attention to the development of individual and social behaviors simultaneously with organizational change. The individual and the organization seem to have a two-way relationship. This is while most current protocols focus on change at one level. This will make it difficult or even impossible to achieve the desired long-term result. This argument seems logical, given the inevitability of some stressors in health-related professions and the impact of individual stressors. Considering the findings of this study on individual and behavioral aspects of workplace stress, a social cognitive approach can be very useful. This approach focuses on the impact of individual attitudes and thoughts on the formation and change of thinking patterns, decision-making, and the way people react and feel. Thus, people learn how destructive thought patterns affect behavior and emotions and identify and change them. In this regard, training and counseling in the field of developing interpersonal communication skills and stress management techniques and problem-solving skills for HCWs, superiors, and managers were found to be useful. Peer-to-peer training, holding training workshops, and sending materials in a virtual manner can be effective. In order to operationalize the conceptual model of the present study, it is proposed to establish a committee including representatives of all stakeholders. This committee can be a nominal group, focus group, or Delphi group, prioritizing the subcategories of each stage of the model. Prioritization should be done based on the criteria of importance, changeability, feasibility, and available resources. In this way, the most important resources and factors affecting stress reduction and the most reasonable interventions will be ranked and selected.

Experiencing the acute conditions of the COVID-19 pandemic led to participants’ deeper understanding of job stress and access to real-world data to design interventions during both epidemics and normal conditions. This valuable impact was observed throughout the findings of this study, particularly in the categories of predisposing and reinforcing factors and developing stress-coping behaviors at work. As far as we know, this is the first qualitative study in Iran that focused on workplace stress among HCWs working in primary health care centers. On the other hand, the samples were heterogeneous because the HCWs were from different primary health care centers. Finally, our study includes the statements and experiences of superiors and managers, in addition to the perspectives of HCWs. This was done to ensure the comprehensiveness of the findings. Another strength is that this study was conducted during the COVID-19 outbreak. Therefore, the findings of one of the most sensitive job conditions of HCWs were included in our study. However, several limitations should be considered in this study. Since, in the primary health care system of Iran, HCWs are mostly women, all participants in our study were women. Therefore, findings related to gender differences in the perception of workplace stress may not be observed in our study. Also, the findings of this study are based on the structure of Iran’s health system; some of our findings may not be meaningful in other countries.

Our study provides guidance to researchers interested in qualitative studies and testing previous theories in other situations or phenomena. On the other hand, the conceptual framework of this study (based on the PPM) could potentially be useful for researchers and policymakers for targeted interventions. In other words, by using our findings (results of the first part of the PPM) and then designing and implementing an intervention based on the second part of the model (PROCEED), empirical studies and comprehensive interventions can be conducted for stress management among HCWs.

## 5. Conclusion

Our study presents a workplace health promotion program as a guide to effective interventions for targeted stress reduction among HCWs. This planning model begins with identifying sources of stress in different aspects, which are the result of participants’ experiences. Then, it provides combined interventions at two levels, aiming at organizational changes and changing behavioral factors affecting workplace stress. In this way, our study emphasizes that workplace stress is a multidimensional issue and that effective control requires intervention across multiple dimensions. Individual and environmental (often organizational) factors are important sources of workplace stress. On the other hand, some factors act as predisposing, enabling, and reinforcing factors that provide the possibility of effective change in individual and environmental causes. In this context, organizational change and educational interventions at individual, social, and organizational levels enable the design of combined, comprehensive, and targeted interventions. In general, for the accurate and valuable design of a mental health promotion program in the workplace, the individual, social, educational, and organizational dimensions of employee stress should be considered together.

The PPM in this study provided the opportunity to identify factors associated with workplace stress and guide its control in HCWs across various dimensions. Additionally, the flexibility of the PPM allowed the researchers in this study to introduce new concepts in addition to the existing ones. Furthermore, Non-occupational factors, Cognitive factors, Supervisory function, Personality characteristics, and Personal context of the employee were identified as new subcategories in the PPM to investigate workplace stress among HCWs. Therefore, we found the PPM very suitable for conceptualizing and designing workplace health promotion programs to manage stress.

## Supporting information

S1 Text Provides details about the PPM and the JD-R model. (DOCX)

S2 Text Interview guide. (DOCX)

S3 Table Detailed participant profiles. (DOCX)
