# Supplementary material for: From source identification to preferential interventions: Determinants of a workplace mental health promotion program to control workplace stress among health care workers based on a qualitative study
Source: PLoS One. 2026 Jan 8;21(1):e0340575. doi: 10.1371/journal.pone.0340575 (PMC12782404; doi:10.1371/journal.pone.0340575)
Supplement: S1 Text — (DOCX) [file pone.0340575.s001.docx]

**Supplementary S1**

**Theoretical Framework**

The PRECEDE-PROCEED model as a planning model has been the cornerstone of the development, implementation and evaluation of many health promotion practices [1,2]. The PRECEDE-PROCEED model to describe problems and devise solutions guides the researcher to choose structures and theories to make effective changes in behavioral and environmental factors related to health [3]. The model has two components. The first component is PRECEDE (Predisposing Reinforcing and Enabling Constructs in Educational / Environmental Diagnosis and Evaluation). The second component is PROCEED (Policy, Regulatory and Organizational Constructs in Educational and Environmental Development) [1,4]. The PRECEDE-PROCEED model has 8 Phase. The first four stages are diagnostic and relate to educational and environmental issues, including: 1) social assessment; 2) epidemiological, behavioral and environmental assessment; 3) environmental educational evaluation and 4) administrative and policy assessment and intervention alignment. The other four steps that involve implementing and evaluating a health promotion intervention are 5) implementation, 6) process evaluation, 7) impact evaluation, and 8) outcome evaluation [3]. Thus, the PRECEDE-PROCEED model considers behavioral, environmental, and social factors for health and has a multi-level, participatory approach to designing health promotion interventions. So we decided to use the model as a health-promoting workplace design framework.

The JD-R model is based on two sets of processes [5]. The first process is stressful in nature, starting with job demands and leading to burnout. Job demands are the physical, psychological, social or organizational aspects of a job that require constant physical-psychological effort and can lead to physiological and psychological costs. The second process is motivational in nature and is driven by the availability of resources and the resulting sense of sacrifice. Job resources are the physical, psychological, social or organizational aspects of the job. Job resources are effective in achieving work goals, personal growth and development, increased motivation and productivity, reducing job demands and physiological and psychological costs [6,7]. On the other hand, when job resources are weak, people experience pessimism about their job, so job resources can be a predictor of work-related stress [7].

1. Green, L.W.; Kreuter, M.. *Health Program Planning*; 4th ed.; McGraw-Hill Education: New York, NY, USA, 2005;

2. Green, L.W.; Kreuter, M.; Deeds, S.G.; Partridge, K.B. Health Education Planning: A Diagnostic Approach. In *Health education planning: a diagnostic approach*; 1980; p. 306.

3. Glanz, K.; Rimer, B.K.; Viswanath, K. Health Behavior: Theory, Research, and Practice. In *Planning Models for Theory-Based Health Promotion Interventions*; Bartholomew, L.K., Markham, C., Mullen, P., Fernández, M.E., Eds.; Jossey-Bass/Wiley, 2015; pp. 372–398 ISBN 1118629000.

4. Glanz, K.; Rimer, B.K.; Viswanath, K. *Health Behavior and Health Education: Theory, Research, and Practice*; John Wiley & Sons, 2008; ISBN 0470432489.

5. Demerouti, E.; Bakker, A.B.; Nachreiner, F.; Schaufeli, W.B. The Job Demands-Resources Model of Burnout. *J. Appl. Psychol.* **2001**, *86*, 499, doi:10.1037/0021-9010.86.3.499.

6. Bakker, A.B.; Demerouti, E. The Job Demands-Resources Model: State of the Art. *J. Manag. Psychol.* **2007**, *22*, 309–328, doi:10.1108/02683940710733115.

7. Bakker, A.B.; Demerouti, E.; Verbeke, W. Using the Job Demands-Resources Model to Predict Burnout and Performance. *Hum. Resour. Manage.* **2004**, *43*, 83–104, doi:10.1002/hrm.20004.
