# Supplementary material for: From source identification to preferential interventions: Determinants of a workplace mental health promotion program to control workplace stress among health care workers based on a qualitative study
Source: PLoS One. 2026 Jan 8;21(1):e0340575. doi: 10.1371/journal.pone.0340575 (PMC12782404; doi:10.1371/journal.pone.0340575)
Supplement: S2 Text — (DOCX) [file pone.0340575.s002.docx]

**S2 Appendix**

**Interview guide**

**Purpose:** This study aimed to identify determinants of a workplace mental health promotion program for stress management among healthcare workers, based on perceptions of employees and superiors and guided by the PRECEDE-PROCEED model.

**Research questions:**

1. Why do HCWs get stressed at work?

2. What is effective in reducing workplace stress?

3. What can be done to create change and intervention?

**Semi-structured interview questions**

- Can you tell me your age, work history, marital status, employment status?
- Please describe your working day.
- If you have experienced a stressful situation at work, describe it?
- In what situations do you experience stress at work?
- What factors cause stress in your workplace these days?
- What do you do when you are stressed?
- What calms you down when you're stressed?
- What things reduce your stress at work?
- How can the organization reduce the stress of health care workers?
- Do you think there is anything else you want to say?

**Focus group questions: (healthcare workers)**

- Under what conditions do you experience stress at work?
- What factors cause stress in your workplace these days?
- What do you do when you are stressed?
- What calms you down when you're stressed?
- What things reduce your stress at work?
- How can the organization reduce the stress of health care workers?
- Do you think there is anything else you would like to say?

**Focus group questions: (superiors)**

- According to your experience, what factors cause health care workers stress and mental pressure?
- What role do superiors play in the stress of caregivers?
- How can the stress of health care workers be reduced?
- How can the organization reduce the stress of health care workers?
- Do you think there is anything else you would like to say?

**Probing questions:**

Probing questions were also used during the interviews and focus groups if needed and according to the situations. Such as; "Please explain more" "How was your situation?" "How did you feel?" "What did you do?", "What did you say?" "What happened next" "What happened in the end?" "How to do it? ...
