# Supplementary material for: From source identification to preferential interventions: Determinants of a workplace mental health promotion program to control workplace stress among health care workers based on a qualitative study
Source: PLoS One. 2026 Jan 8;21(1):e0340575. doi: 10.1371/journal.pone.0340575 (PMC12782404; doi:10.1371/journal.pone.0340575)
Supplement: S3 Table — (DOCX) [file pone.0340575.s003.docx]

S3 Table

**Table 1**

*Demographic Characteristics and Work Experience of Health Care Workers*

| Participant | Gender | Age | Marital Status | Parent | Occupation | Employment Contract | Work Experience |
| --- | --- | --- | --- | --- | --- | --- | --- |
| P1 | Woman | 40 | Married | Mother | Health care worker | Permanent contract | 15 |
| P2 | Woman | 45 | Single | - | Health care worker | Permanent contract | 23 |
| P3 | Woman | 38 | Married | Mother | Health care worker | Permanent contract | 12 |
| P4 | Woman | 36 | Married | Mother | Health care worker | Permanent contract | 9 |
| P5 | Woman | 30 | Married | Mother | Foreman | Permanent contract | 10 |
| P6 | Woman | 28 | Married | Mother | Health care worker | fixed-term contract | 1 |
| P7 | Woman | 27 | Single | - | Foreman | fixed-term contract | 4 |
| P8 | Woman | 25 | Married | - | Foreman | fixed-term contract | 2 |
| P9 | Woman | 27 | Married | - | Foreman | fixed-term contract | 4 |
| P10 | Woman | 40 | Married | Mother | Foreman | fixed-term contract | 7 |
| P11 | Woman | 42 | Married | Mother | Foreman | Permanent contract | 18 |
| P12 (FG*) | Woman | 25 | Single | - | Foreman | fixed-term contract | 2 |
| P13 (FG) | Woman | 39 | Married | Mother | Foreman | fixed-term contract | 7 |
| P14 (FG) | Woman | 30 | Married | - | Foreman | fixed-term contract | 5 |
| P15 (FG) | Woman | 42 | Married | Mother | Foreman | fixed-term contract | 3 |
| P16 (FG) | Woman | 27 | Single | - | Foreman | fixed-term contract | 3 |
| P17 (FG) | Woman | 37 | Married | Mother | Foreman | Permanent contract | 10 |
| P18 (FG) | Woman | 35 | Single | - | Foreman | Permanent contract | 5 |
| P19 (FG) | Woman | 35 | Married | - | Foreman | fixed-term contract | 3 |
| P20 (FG) | Woman | 31 | Married | Mother | Foreman | fixed-term contract | 4 |
| P21 (FG) | Woman | 28 | Single | - | Foreman | fixed-term contract | 4 |

FG*: Focus Group

**Table 2**

*Demographic Characteristics and Work Experience of Superiors*

| Participant | Gender | Age | Occupation | Work Experience |
| --- | --- | --- | --- | --- |
| P22 (FG) | Woman | 48 | Supervisor | 7 |
| P23 (FG) | Woman | 37 | Supervisor | 6 |
| P24 (FG) | Woman | 38 | Supervisor | 10 |
| P25 (FG) | Woman | 36 | Supervisor | 7 |
| P26 (FG) | Woman | 30 | Supervisor | 5 |
| P27 (FG) | Woman | 36 | Supervisor | 6 |
| P28 (FG) | Woman | 47 | Supervisor | 22 |
| P29 (FG) | Woman | 48 | Supervisor | 11 |
| P30 (FG) | Man | 46 | Supervisor | 17 |
| P31 (FG) | Man | 45 | Manager | 20 |
| P32 (FG) | Man | 47 | Manager | 20 |

FG: Focus Group

|  |  |
| --- | --- |
